# Impacts of the COVID-19 pandemic on subjective wellbeing in the Middle East and North Africa: A gender analysis

**DOI:** 10.1371/journal.pone.0286405

**Published:** 2023-05-31

**Authors:** Maia Sieverding, Caroline Krafft, Irene Selwaness, Alexandra Abi Nassif

**Affiliations:** 1 Faculty of Health Sciences, Department of Health Promotion and Community Health, American University of Beirut, Beirut, Lebanon; 2 Department of Economics and Political Science, St. Catherine University, St. Paul, Minnesota, United States of America; 3 Faculty of Economics and Political Science, Cairo University, Giza, Egypt; Ladoke Akintola University of Technology Teaching Hospital: LAUTECH Teaching Hospital, NIGERIA

## Abstract

The pathways through which the COVID-19 pandemic has impacted population mental health are potentially gendered. Little research has explored these pathways in low- and middle-income country contexts, such as in the Middle East and North Africa (MENA) region, where socioeconomic roles are highly gendered. To address this gap, we examine the relationships between pandemic-related socioeconomic changes and subjective wellbeing in the MENA region. Our core hypothesis is that the COVID-19 pandemic affected men and women’s subjective wellbeing differently in part because these effects were mediated by gendered socioeconomic roles. We exploit multiple waves of longitudinal, nationally-representative phone survey data across Egypt, Jordan, Morocco, Sudan, and Tunisia. The data were collected between November 2020 and August 2021 and include 32,296 observations of 20,256 unique individuals. Mental health is measured through the WHO-5 subjective wellbeing scale. Our key independent variables capture pandemic-related employment loss, income loss, experience of limitations on food access, enrollment of children in alternative schooling modalities, and receipt of formal and informal transfers. We find significantly worse subjective wellbeing for women in Egypt and Morocco during the pandemic, but not the three other countries. There were negative associations between employment and income loss during the pandemic and subjective wellbeing, but not gender-differentiated ones. In contrast, high levels of limitations on food access were associated with worse mental health for men than women. Receipt of transfers generally did not have any association with subjective wellbeing. Further research is needed into how social assistance programs implemented in response to pandemics may be designed so as to address the negative mental health consequences of such events.

## Introduction

Globally, the COVID-19 pandemic has had substantial negative mental health effects in the general population [1]. The pathways through which the pandemic and associated social distancing measures have affected mental health are numerous and gendered. Gendered social roles that are associated with chronic stress, such as caregiving responsibilities, tend to be reinforced during health crises, placing an added burden on women. Gender roles may also mediate how men and women react to stressors exacerbated by the pandemic, such as loss of social support and financial insecurity [2].

The Middle East and North Africa (MENA) region is characterized both by highly gendered socioeconomic roles [3] and by a substantial burden of poor mental health that is more severe among women than men [4]. There continues to be strong adherence to a ‘male breadwinner-female caregiver’ household model in MENA [3,5]. This translates into the region having, prior to the pandemic, the highest female-to-male ratio of time spent on unpaid care work [6] and the lowest female labor force participation rates [7] of any world region. There has been very little attention to how these gendered socioeconomic roles may contribute to the mental health burden in the region.

A robust literature has emerged on the gendered socioeconomic impacts of the COVID-19 pandemic [8], and the implications of these impacts for mental health [2,9–11]. However, much of this literature comes from high-income countries (HICs) where gendered patterns of pre-pandemic socioeconomic roles were distinct. The MENA region is an interesting context within which to explore how the effects of the pandemic have taken shape against a background of relatively rigid gender roles and a large gender gap in economic empowerment. Furthermore, very little is known about how pandemic-related changes have affected mental health in the region.

Consistent with global literature, the small literature on mental health in MENA during the COVID-19 pandemic has found negative population-level mental health effects [12] and that women experienced higher levels of psychological stress than men [13,14]. However, these studies are based predominantly on cross-sectional, convenience samples and focus on sociodemographic predictors of poor mental health. The results therefore cannot be generalized to the national level and are not grounded in a gender perspective on how the pandemic has affected mental health.

In this paper, we use data from a collection of nationally representative, longitudinal surveys, the COVID-19 MENA Monitor (CMM), to explore how gender has mediated the relationship between COVID-19-related socioeconomic changes and mental health in the MENA region. We measure mental health through the WHO-5 subjective wellbeing scale. Subjective wellbeing and mental health are closely related, but subjective wellbeing focuses on the positive dimension of mental health, often measured through life satisfaction or feelings of happiness [15]. The WHO-5 seeks to measure the affective dimensions of wellbeing, or people’s experience of happiness and positive emotional states [15]. Since the global [1,16] and small regional [12–14] literatures on mental health during the COVID-19 pandemic use a wide range of measures, many of which in fact capture subjective wellbeing, we use the terms interchangeably. The paper contributes to the global literature on the pandemic’s socioeconomic and health impacts with a perspective from little-studied low- and middle-income country (LMIC) contexts, namely Egypt, Jordan, Morocco, Tunisia, and Sudan. It also contributes to the theorization of how gender roles mediate the health impacts of crises.

## Theoretical framework and hypotheses

The core premise of our theoretical framework is that the COVID-19 pandemic has affected men’s and women’s subjective wellbeing differently in part because these effects are mediated by gendered socioeconomic roles [2]. Our hypotheses regarding how gendered roles mediate the effects of specific types of socioeconomic changes are based on the limited previous literature on the determinants of mental health in the MENA region as well as the global literature on the impact of the COVID-19 pandemic on mental health.

Given that women in the MENA region suffered from a greater burden of poor mental health prior to the pandemic [4], and that the global literature has demonstrated worse mental health outcomes among women during the pandemic than men [1,16], our first hypothesis is that this gender differential held in MENA during the pandemic as well.

***H1***: *Women in MENA experienced worse subjective wellbeing during the COVID-19 pandemic than men*.

We then turn to hypotheses regarding specific socioeconomic changes that occurred widely during the pandemic, namely unemployment, loss of income, changes in food security, receipt of social assistance from programs aiming to mitigate the pandemic’s economic impacts, and changes in children’s schooling modalities.

The pandemic and associated lockdown measures had severe impacts on labor markets around the world, including job losses in many sectors [17]. Globally, unemployment during the pandemic was associated with higher odds of psychological distress, including anxiety and depression [16]. In one of few studies from an LMIC, employment continuity during the pandemic was also found to be protective of mental health in South Africa. The impacts of job loss on mental health in this study were not, however, gendered [18].

Female labor force participation in MENA is low [19]. Women also tend to be concentrated in public sector employment, which was generally protected from job losses during the pandemic [20]. Previous literature from the region found negative effects of unemployment only on men’s mental health, likely due to gender norms that make employment an expectation for men but not for women [21]. Therefore, with respect to employment, our hypothesis is:

***H2*:** *Loss of employment during the pandemic had a negative impact on men’s subjective wellbeing but not women’s*.

The labor market challenges caused by the pandemic led to loss of or instability in income among many households. Studies from contexts including China [22] and the United States [23] have found that income loss during the pandemic was associated with worse mental health. Income loss is in turn one of several factors that may have affected household food security during the pandemic. Mobility restrictions and disruptions to supply chains in many countries also contributed to reduced access to and availability of food and increases in prices that negatively affected food security [24]. A robust global literature that, in contrast to our other domains, includes a number of studies from LMIC contexts, has consistently demonstrated a negative association between food insecurity and mental health during the pandemic [23,25–27], however these studies do not consider the potential mediating role of gender.

In their traditional gendered role as primary caretakers in the household, women may internalize worry over resource scarcity [2] and lack of food specifically [10] such that their mental health is more affected by these factors than that of men. On the other hand, inability to provide for household needs may threaten men’s traditional role as breadwinner, leading to adverse mental health effects. One study in South Africa found that the negative mental health effects of food insecurity during the pandemic were greater for men than women [10]. In the MENA region, men are expected to uphold this role of breadwinner, which is central to notions of masculinity [3,5]. However, women are usually the ones who purchase food and manage the daily household budget [28], and so may be more exposed to daily stressors regarding resource constraints. We therefore hypothesize that:

***H3***: *Loss of income and food insecurity during the pandemic had a negative impact on subjective wellbeing for both women and men*.

In an attempt to counteract some of the negative economic effects of the COVID-19 pandemic, governments around the world implemented temporary unemployment insurance, cash transfer, or other social assistance programs. These programs were extremely heterogenous in their targeting, amounts disbursed, and duration (see S1 File, for a summary of the social assistance programs implemented in the study countries). Households may also have increased reliance on informal transfers from relatives, community members or charitable organizations to make ends meet during the pandemic [29–31].

The relatively small literature on the mental health effects of receiving such transfers has shown mixed results and has not focused on gender as a potential mediating factor [23,26,27]. In the only study we identified from an LMIC, in South Africa receipt of social grants was weakly positively associated with mental health during the pandemic, but the effect appeared to be short-lived [18]. There are no studies from the MENA region on the mental health effects of receiving pandemic-related transfers. Therefore, based on the general evidence that receipt of cash transfers positively impacts mental health [32], we hypothesize that:

***H4***: *Receipt of formal and informal transfers is protective of subjective wellbeing for both women and men*.

Finally, households with children were impacted by the worldwide closure of schools and nurseries as a precaution against the spread of COVID-19. In many countries, including those covered by this study, children spent varying periods of time without schooling or completing schooling through alternative modalities such as online learning (see S1 File, for a summary of school closures in the study countries). The mental health impacts on parents of the resulting burden of additional childcare, and how it was divided within the household, often depended on interactions with employment and sex [9,11].

The overwhelming majority of childcare is performed by women in the MENA region [3]. Provision of tutoring and academic support were also very common in the region even prior to the pandemic, tasks that are also often performed by mothers [33]. We therefore expect that most of the additional care- and school-work burden resulting from pandemic school closures was taken up by women, with the result that:

***H5*:** *In households where children were attending alternative schooling modalities*, *women experienced worse subjective wellbeing but not men*.

## Methods

### Data

We use data from the CMM, a collection of nationally representative, longitudinal surveys that aimed to track the economic impact of COVID-19 in the MENA region [34]. The surveys were conducted by phone over five waves between November 2020 and August 2021 in Egypt, Jordan, Morocco, Sudan, and Tunisia. Between two and four survey waves were conducted in each country, starting with an initial sample of about 2,000 individuals per country who were tracked over time (see S1 Table). A refresher sample was added as needed to combat attrition, which was relatively high [29–31]; the five-country dataset contains 32,296 observations of 20,256 unique individuals.

The universe for the survey was mobile phone users aged 18–64. Random digit dialing, with up to three attempts to complete the survey, was used to select the sample. Weights account for both the sampling strategy and initial non-response by observable characteristics relative to the population of 18-64-year-old phone owners in nationally representative pre-COVID-19 in-person surveys. Attrition models were used to construct weights for panel respondents.

The COVID-19 MENA Monitor data collection underwent IRB review and approval at St. Catherine University (#1415). All participants gave oral informed consent at the start of the survey. Participants were informed that any personal information would be kept strictly confidential, and gave separate, additional consent for follow-up waves of data collection. The purpose of the research, to understand the impact of the COVID-19 outbreak in the region, was explained to participants as part of the consent process. As secondary analysis of de-identified, publicly available data, the analysis presented in this paper was not subject to additional IRB review. Our use and storage of data was in line with the requirements of the data providers, including use only for scholarly purposes, secure storage of the data, and proper citation of the data.

### Outcome measure

Our outcome, subjective wellbeing, is measured through the World Health Organization Well-Being Index (WHO-5). The WHO-5 is built on a positive approach to mental health and correspondingly seeks to measure positive emotional states [15]. Respondents are posed five statements about their life over the past two weeks: “I have felt cheerful and in good spirits;” “I have felt calm and relaxed;” “I have felt active and vigorous;” “I woke up feeling fresh and rested;” and “my daily life has been filled with things that interest me.” Response choices range from “all of the time” (five) to “none of the time” (zero). The raw score of 0–25 is multiplied by four to convert to a scale that ranges from zero (minimal wellbeing) to 100 (maximum wellbeing) [35]. The WHO-5 has been widely used internationally and has high validity across different sociocultural contexts [35]. In the CCM surveys, the scale showed acceptable consistency, with an overall Cronbach’s alpha of 0.78 across the five countries. The Cronbach’s alpha at the country level was 0.74 for Jordan, 0.89 for Morocco, 0.80 for Sudan, 0.68 for Tunisia and 0.70 for Egypt.

### Measures of COVID-19 socioeconomic changes

Our key independent variables capture experiences of job loss, income changes, changes in access to food, receipt of transfers, and children’s alternative schooling modalities during the COVID-19 pandemic. S2 Table in the supplemental materials provides summary statistics on experiencing these changes by country and sex. Changes in labor force status were generated as a categorical variable by comparing the respondent’s labor force status in February 2020, i.e. immediately prior to the start of the pandemic, with their labor force status at the time of the survey. The variable is coded as stayed employed, stayed not employed, left employment, or entered employment. Non-employed statuses cover both unemployment and being out of the labor force.

For income changes, respondents were asked how their total household income in the month prior to the survey compared to the month of February 2020, with response choices of “decreased by more than 25%,” “decreased by 1–25%,” “stayed the same,” “increased by 1–25%” and “increased by more than 25%.” We collapsed the two increased categories into one due to small cell sizes and otherwise retained the variable as is. This question was asked differently in Sudan due to very high rates of inflation during the period of the survey. Respondents were asked to compare their ability to pay for goods and services in the past month as compared to February 2020, with response choices of “much worse,” “somewhat worse,” “about the same,” “somewhat better,” and “much better.” We mapped each response category (from worst to best) to the corresponding categories in the main income question (from decreased the most to increased the most).

To measure changes in food access, respondents were asked whether, in the seven days prior to the survey, anyone in the household experienced any of the following: (1) difficulties in going to food markets due to mobility restrictions; (2) being unable to buy usual quantities of food because of shortages; (3) being unable to buy usual quantities of food because of price increases; (4) being unable to buy usual quantities of food because of a decline in income; and (5) having to reduce the number of meals and/or portion sizes consumed. Our food access measure is constructed as a count variable of the number of limitations on food access the household experienced, that ranges from zero to five.

Receipt of transfers is measured through three separate binary variables. The first captures whether the household received any regular (not pandemic-related) form of cash or food (e.g. ration cards) assistance from the government or, in the countries with humanitarian situations, UN agencies. This measure did not vary across survey waves for individuals with multiple observations. The second measure captured whether the respondent reported receiving a temporary form of government cash transfer related to the pandemic (e.g. non-regular cash payments or unemployment payments) in the month prior to the survey. The third measure captured whether the respondent reported receiving “food, cash or other support” in the month prior to the survey from a relative or religious, political or charity organization. We term this as receipt of social support.

Finally, for respondents living in households with school-age children, we include two binary measures of schooling modalities that capture (1) whether any child in the household was engaged in an alternative school modality (online schooling, educational television, or official written materials) at the time of the survey; and (2) whether the respondent was helping any child in the household with their schoolwork.

### Sociodemographic controls

Our key sociodemographic factor of interest is the respondent’s sex. In addition, we include controls for potential sociodemographic correlates of subjective wellbeing during the pandemic [1,16] and measures of household structure that may mediate the relationship between socioeconomic changes and wellbeing. The sociodemographic controls consist of age, urban/rural residence, education (categorized as: less than basic; basic (equivalent to 9^th^ grade in most countries); secondary; or tertiary), and marital status (categorized as: never married; married; or widowed/divorced). For Jordan, we also include a variable for whether the respondent is a refugee, which includes both Palestinian and Syrian refugees. The household-level variables are total household size, a binary variable for whether the household contains any children under age six, and a binary variable for whether there are any school-age children in the household. S3 Table summarizes these characteristics of the survey respondents.

### Analysis

We first present the trend in the mean WHO-5 score, by country and sex, over the study period. We then build our multivariate models stepwise. First, to test H1, we estimate an ordinary least squares regression model that includes only sex and the sociodemographic and household structure variables. We then add to this model the measures of COVID-19 related changes that apply to all respondents (labor market status, income, food access, and transfers). The measure of each socioeconomic change is interacted with sex to test hypotheses H2-H4. Finally, to test H5, we add to the model our measures of schooling modalities, again interacted with sex; this model is limited to respondents in households with school age children (N = 17,937).

At each step, we estimate the model both on the pooled five-country sample and for each country individually. The pooled models include additional controls for country, survey wave and country-wave interactions. The country-specific models include controls for survey wave. All analyses are conducted using sample weights and standard errors are clustered at the individual level. As a robustness check, we also exploit the longitudinal structure of the data to estimate individual fixed-effects models for the models testing hypotheses H2-H4 and H5. In this fixed-effects analysis, all time-invariant characteristics of respondents necessarily drop out, including receipt of regular government transfers.

## Results

### Subjective wellbeing in MENA during the COVID-19 pandemic

**Fig 1:**
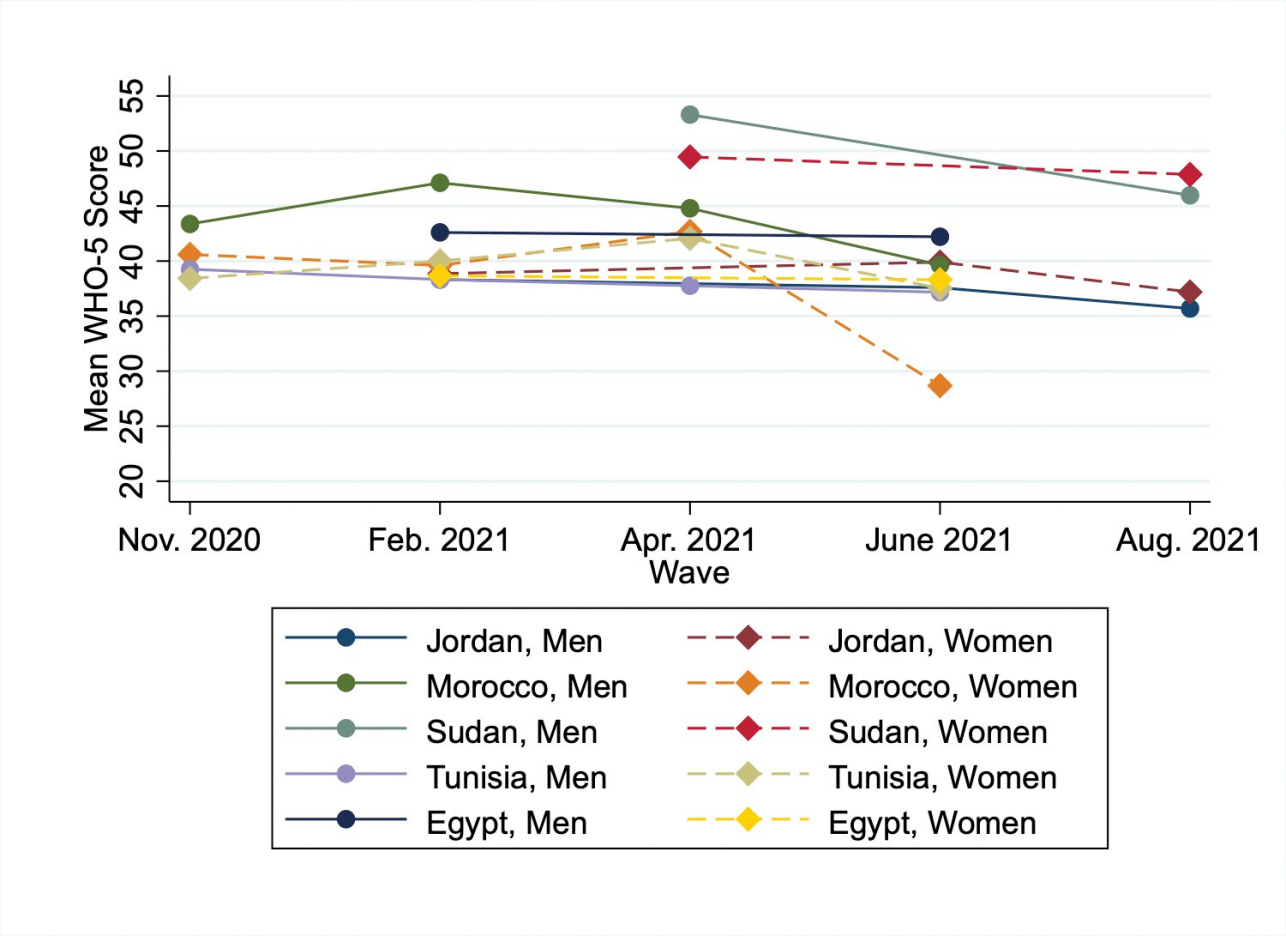
Mean WHO-5 score by wave, country and sex. Source: Constructed by the authors based on CMM data.

The mean WHO-5 score did not exceed 50 for any country-sex group during the study period, except for men in Sudan during the April 2021 survey wave (Fig 1). Respondents in Sudan generally had the highest WHO-5 scores, followed by men in Egypt and Morocco. In most countries, the general trend in the WHO-5 was downward, albeit slightly, over the study period. Particularly large changes were observed in Morocco, where the mean WHO-5 score among men increased between November 2020 and February 2021 before declining again through June 2021, and the score for women declined by over 10 points between April and June 2021. Women in Tunisia and both men and women in Jordan also saw declines in the mean WHO-5 score in the final waves conducted in each country in summer 2021.

### Models of subjective wellbeing and sex

In the pooled country model testing H1, women experienced significantly lower WHO-5 scores, by 1.32 points (p<0.05), as compared to men (Table 1; see S4 Table for results for other sociodemographic controls). However, this result was driven by gender differences in only two countries: Morocco, where women scored 5.14 points lower on the WHO-5 than men (p<0.001) and Egypt, where women scored 3.18 points lower (p<0.01). There was no significant gender difference in subjective wellbeing in the other three countries

**Table 1:** Associations (OLS regression coefficients) between subjective wellbeing and sex, controlling for sociodemographic, and household structure variables, all respondents (H1).

	(1)	(2)	(3)	(4)	(5)	(6)
Outcome variable: Subjective well-being	Pooled	Jordan	Morocco	Sudan	Tunisia	Egypt
**Sex (ref: male)**						
Female	-1.32*	1.19	-5.14***	0.63	0.48	-3.18**
	[-2.56 - -0.09]	[-0.65–3.04]	[-7.94 - -2.34]	[-4.03–5.29]	[-2.01–2.97]	[-5.21 - -1.16]
**Includes controls for country-wave interaction**	YES	N/A	N/A	N/A	N/A	N/A
**Includes sociodemographic controls**	YES	YES	YES	YES	YES	YES
**Observations**	32,296	7,625	8,120	4,401	8,143	4,007
**R-squared**	0.03	0.04	0.03	0.05	0.03	0.02

Notes: 95% confidence intervals in brackets

* p<0.05

** p<0.01

***p<0.00.

### Associations between COVID-19 related socioeconomic changes and subjective wellbeing

We now turn to the models that add COVID-19 related socioeconomic changes, interacted with sex, to the sociodemographic predictors (Table 2). Staying not employed was associated with a 3.33-point lower WHO-5 score (p<0.01) and leaving employment with a 3.91-point lower score (p<0.001). At the country level, the negative association with staying not employed was seen only in Sudan. By contrast, the result for leaving employment was consistent across all countries, although it was not significant in Jordan or Tunisia. A positive association between entering employment and subjective wellbeing was seen only in Tunisia. In relation to hypothesis H2, the only significant interaction was between sex and staying not employed in the pooled model (3.72 points; p<0.05); whereas men who stayed not employed had worse subjective wellbeing, for women this was not the case.

**Table 2:** Associations (OLS regression coefficients) between subjective wellbeing and sex, and COVID-19 related changes, all respondents (H2-H4).

	(1)	(2)	(3)	(4)	(5)	(6)
Outcome variable: Subjective well-being	Pooled	Jordan	Morocco	Sudan	Tunisia	Egypt
**Sex (ref: male)**						
Female	-4.82**	-1.81	-14.31***	3.87	2.83	-6.23*
	[-7.95 - -1.69]	[-6.64–3.03]	[-22.08 - -6.55]	[-13.78–21.53]	[-3.33–8.98]	[-12.33 - -0.13]
**Labor market change vs. Feb. 2020 (ref: stayed employed)**						
Stayed not employed	-3.33**	-0.60	-3.89	-9.58*	0.05	-0.97
	[-5.72 - -0.94]	[-3.73–2.53]	[-9.05–1.27]	[-17.12 - -2.04]	[-5.17–5.27]	[-4.86–2.91]
Left employment	-3.91***	-0.83	-6.21**	-7.22*	-2.57	-3.99*
	[-6.11 - -1.71]	[-4.56–2.90]	[-10.30 - -2.13]	[-13.48 - -0.95]	[-5.33–0.19]	[-7.44 - -0.53]
Entered employment	-0.30	2.86	0.29	-7.80	4.89*	-4.55
	[-3.17–2.57]	[-1.60–7.33]	[-5.60–6.18]	[-15.82–0.22]	[0.01–9.77]	[-9.34–0.24]
Stayed not employed # female	3.72*	-1.73	7.34	4.82	2.81	1.09
	[0.37–7.06]	[-6.65–3.18]	[-0.50–15.19]	[-10.14–19.78]	[-3.48–9.10]	[-4.43–6.61]
Left employment # female	1.95	-2.61	8.34	-2.14	1.66	0.60
	[-3.33–7.23]	[-9.25–4.02]	[-1.02–17.70]	[-22.78–18.49]	[-9.81–13.13]	[-5.78–6.97]
Entered employment # female	1.07	-4.07	-4.51	4.51	0.01	6.34
	[-3.35–5.49]	[-11.27–3.13]	[-14.74–5.72]	[-11.30–20.33]	[-7.02–7.03]	[-1.17–13.84]
**Limitations in food access (ref: no change)**						
1 limitation	-8.25***	-6.48***	-13.24***	-3.34	-2.40	-6.91***
	[-10.48 - -6.02]	[-9.81 - -3.14]	[-18.10 - -8.37]	[-12.38–5.70]	[-7.16–2.36]	[-9.98 - -3.83]
2 limitations	-11.59***	-12.24***	-13.37***	-1.74	-10.50***	-10.70***
	[-13.89 - -9.28]	[-15.55 - -8.93]	[-18.28 - -8.46]	[-10.53–7.05]	[-14.77 - -6.23]	[-14.18 - -7.23]
3 limitations	-13.62***	-13.80***	-16.27***	-3.84	-12.06***	-10.39***
	[-15.96 - -11.29]	[-17.27 - -10.33]	[-21.73 - -10.81]	[-12.57–4.89]	[-16.02 - -8.10]	[-13.76 - -7.02]
4 limitations	-14.63***	-11.58***	-15.49***	-5.39	-15.07***	-10.58***
	[-17.22 - -12.05]	[-16.14 - -7.01]	[-21.54 - -9.43]	[-14.72–3.94]	[-19.29 - -10.84]	[-15.03 - -6.13]
5 limitations	-19.23***	-18.44***	-20.58***	-4.49	-17.13***	-15.96***
	[-21.93 - -16.52]	[-22.84 - -14.04]	[-28.93 - -12.22]	[-15.90–6.92]	[-21.42 - -12.83]	[-20.29 - -11.64]
1 limitation # female	1.14	-0.21	4.75	-0.86	-3.49	2.57
	[-2.19–4.46]	[-4.98–4.56]	[-2.77–12.28]	[-13.21–11.49]	[-11.47–4.48]	[-2.56–7.70]
2 limitations # female	1.78	3.92	4.04	-7.65	-2.98	3.74
	[-1.55–5.11]	[-0.79–8.62]	[-3.59–11.67]	[-20.54–5.25]	[-9.72–3.76]	[-1.44–8.91]
3 limitations # female	3.83*	4.08	5.70	2.00	-2.68	1.20
	[0.48–7.18]	[-0.69–8.85]	[-2.51–13.90]	[-11.02–15.02]	[-9.39–4.02]	[-4.08–6.49]
4 limitations # female	3.44	3.46	3.95	-1.71	-1.82	0.13
	[-0.22–7.09]	[-3.39–10.31]	[-5.27–13.18]	[-14.88–11.47]	[-8.76–5.12]	[-7.12–7.37]
5 limitations # female	4.94*	12.10***	5.01	9.15	-5.32	2.82
	[1.00–8.88]	[5.13–19.07]	[-6.68–16.71]	[-8.83–27.13]	[-12.61–1.96]	[-3.91–9.55]
**Income changes vs. Feb. 2020 (ref: stayed the same)**						
Decreased by more than 25%	-3.83***	-6.07***	-4.61*	-8.84*	-1.85	-4.07*
	[-5.71 - -1.95]	[-8.99 - -3.14]	[-8.90 - -0.31]	[-15.95 - -1.74]	[-5.07–1.36]	[-7.30 - -0.84]
Decreased by 1–25%	-2.07*	-4.12**	-2.47	-5.97	-0.63	-2.89*
	[-4.02 - -0.12]	[-7.07 - -1.17]	[-7.87–2.93]	[-13.76–1.83]	[-3.51–2.26]	[-5.60 - -0.17]
Increased	2.63	1.73	0.08	6.07	2.89	-2.42
	[-0.11–5.37]	[-2.08–5.54]	[-9.75–9.91]	[-2.95–15.09]	[-0.93–6.71]	[-6.39–1.56]
Decreased by more than 25% # female	-0.02	3.30	2.87	-3.19	-0.97	3.51
	[-2.75–2.70]	[-0.80–7.40]	[-3.65–9.38]	[-15.04–8.67]	[-5.57–3.63]	[-1.25–8.28]
Decreased by 1–25% # female	1.00	3.86	1.91	-3.55	0.15	2.62
	[-1.78–3.77]	[-0.16–7.88]	[-6.19–10.02]	[-16.46–9.36]	[-4.56–4.86]	[-1.51–6.76]
Increased # female	-1.12	2.39	1.48	-5.81	-4.04	2.77
	[-4.97–2.73]	[-3.38–8.15]	[-11.39–14.36]	[-18.74–7.12]	[-10.02–1.94]	[-5.34–10.88]
**Receipt of transfers**						
Regular government support	-0.31	-0.77	0.40	5.80	-1.28	-0.48
	[-2.09–1.46]	[-3.39–1.85]	[-5.08–5.89]	[-0.91–12.50]	[-5.49–2.93]	[-3.28–2.33]
Regular government support # female	0.55	2.89	-0.06	-9.85*	0.65	-0.36
	[-1.74–2.84]	[-0.70–6.49]	[-7.83–7.71]	[-19.12 - -0.58]	[-5.23–6.52]	[-4.70–3.99]
Temporary government support	-0.28	-2.33	-0.84	-1.32	-4.52	-0.38
	[-3.44–2.88]	[-7.31–2.66]	[-7.41–5.73]	[-8.28–5.64]	[-11.25–2.21]	[-5.06–4.29]
Temporary government support # female	1.57	3.19	4.35	-0.58	9.06	3.73
	[-3.03–6.18]	[-3.11–9.49]	[-6.32–15.02]	[-11.92–10.76]	[-1.10–19.22]	[-5.76–13.23]
Last month social support	-0.89	0.19	1.46	-3.47	0.95	0.18
	[-3.54–1.77]	[-3.89–4.27]	[-5.14–8.05]	[-9.61–2.67]	[-4.00–5.89]	[-3.86–4.22]
Last month social support # female	3.86	-0.06	3.94	10.01	0.82	2.24
	[-0.33–8.06]	[-5.27–5.16]	[-8.12–15.99]	[-0.23–20.26]	[-6.65–8.29]	[-3.87–8.36]
Constant	53.46***	48.59***	55.09***	60.58***	55.08***	54.88***
	[49.03–57.88]	[41.93–55.26]	[47.19–63.00]	[46.54–74.63]	[48.36–61.79]	[48.19–61.58]
Includes sociodemographic controls	YES	YES	YES	YES	YES	YES
Includes controls for survey wave	YES	YES	YES	YES	YES	YES
Includes controls for country	YES	N/A	N/A	N/A	N/A	N/A
Includes controls for country-wave interaction	YES	N/A	N/A	N/A	N/A	N/A
Observations	32,275	7,625	8,099	4,401	8,143	4,007
R-squared	0.09	0.10	0.09	0.13	0.12	0.08

Notes: 95% confidence intervals in brackets

* p<0.05

** p<0.01

***p<0.001.

Limitations in food access during the pandemic were associated with large and significant decreases in subjective wellbeing in a dose-response manner. In the pooled model, this ranged from an 8.25-point lower score among those experiencing one limitation (p<0.001) to a 19.23-point lower score among those experiencing all five types of limitations (-16.52; p<0.001). Although the magnitude of the coefficients varied, this pattern held in all countries except Sudan, where food access limitations were not significantly associated with WHO-5 scores. In terms of H3, differences by sex were seen in the pooled model at higher numbers of limitations; women facing three and five limitations experienced smaller declines in wellbeing as compared to men (the coefficient for four limitations was similarly positive but not significant). At the country level, the interaction between food access limitations and sex was positive and significant only for women in Jordan experiencing five limitations.

Decreases in monthly income, as compared to February 2020, were also associated with worse subjective wellbeing in a dose-response manner. Respondents who reported that their household income decreased by more than 25% experienced a 3.83-point lower WHO-5 score (p<0.001) in the pooled model and those whose income decreased by 1–25% a 2.07-point lower score (p<0.05). The coefficient for increased income was positive but insignificant. At the country level, the association between the largest income decline and worse subjective wellbeing held for all countries except Tunisia, where the coefficient was negative but insignificant. For income declines of 1–25%, the coefficients were negative for all countries but only significant in Jordan and Egypt. Testing H3, none of the interactions with sex were significant.

Turning to H4, receipt of transfers was not significantly associated with subjective wellbeing for any of the transfer types or in any country. The only significant interaction was for women in Sudan who received regular government transfers, who experienced worse subjective wellbeing as compared to men. This may be a case of reverse causality, where the women targeted for regular government transfers were particularly disadvantaged.

Finally, helping children with schoolwork was positively associated with subjective wellbeing in the pooled model (Table 3; 3.03 points; p<0.01) and in Jordan (3.84 points; p<0.01), but the interaction effect in Jordan was negative (-5.71 points; p<0.01) indicating that while men experienced higher wellbeing when helping with schoolwork women experienced lower wellbeing. By contrast, the interaction with sex was positive in Egypt (5.22 points; p<0.01). Children being engaged in an alternative schooling modality was associated with better subjective wellbeing in Sudan (10.95 points; p<0.01) but no other results related to alternative schooling were significant. Results were relatively robust in the fixed-effect models (see S2 File).

**Table 3:** Associations (OLS regression coefficients) between subjective wellbeing and changes in schooling modalities, respondents with school aged children (H5).

	(1)	(2)	(3)	(4)	(5)	(6)
	Pooled	Jordan	Morocco	Sudan	Tunisia	Egypt
**Sex (ref: male)**						
Female	-6.88**	-4.12	-17.02***	5.95	-2.35	-8.88
	[-12.09 - -1.67]	[-13.22–4.97]	[-26.70 - -7.34]	[-16.48–28.38]	[-15.32–10.62]	[-21.11–3.34]
**Respondent helps with school work**	3.03**	3.81*	5.23	4.57	1.30	1.05
	[1.05–5.01]	[0.78–6.84]	[-0.75–11.20]	[-2.48–11.63]	[-1.83–4.43]	[-2.09–4.18]
Respondent helps with school work # female	-0.85	-5.68**	-2.84	-8.09	1.81	5.22*
	[-3.74–2.03]	[-9.86 - -1.49]	[-10.99–5.30]	[-18.50–2.32]	[-3.03–6.66]	[0.19–10.26]
**Children in alternative schooling modality**	2.00	3.85	-2.12	10.95**	-5.73	4.74
	[-0.82–4.82]	[-1.46–9.16]	[-6.55–2.31]	[4.19–17.71]	[-12.32–0.86]	[-1.59–11.07]
Children in alternative schooling modality # female	2.71	4.65	-3.59	0.28	7.47	3.85
	[-1.28–6.71]	[-2.51–11.82]	[-10.14–2.96]	[-10.41–10.96]	[-2.39–17.32]	[-5.84–13.54]
Constant	48.81***	41.42***	56.19***	51.53***	55.44***	46.87***
	[41.91–55.70]	[30.59–52.26]	[44.81–67.56]	[34.33–68.73]	[44.16–66.73]	[35.71–58.03]
Includes sociodemographic controls	YES	YES	YES	YES	YES	YES
Includes controls for other COVID-19 changes	YES	YES	YES	YES	YES	YES
Includes controls for survey wave	YES	YES	YES	YES	YES	YES
Includes controls for country	YES	N/A	N/A	N/A	N/A	N/A
Includes controls for country-wave interaction	YES	N/A	N/A	N/A	N/A	N/A
Observations	17,937	4,787	4,548	2,174	4,008	2,420
R-squared	0.10	0.11	0.10	0.15	0.14	0.12

Notes: 95% confidence intervals in brackets

* p<0.05

** p<0.01

***p<0.001.

## Discussion

Using comparable data from five countries in MENA, we found a high burden of poor wellbeing during the COVID-19 pandemic. The mean WHO-5 score for men ranged from 36–53 and for women 29–49 across waves and countries. Although nationally representative pre-pandemic data on subjective wellbeing in the region are limited, these scores are generally worse than in Egypt in 2018 [36] and Palestine in 2012–13 [37]. Given the limited mental health care services available in the region and the weakness of health systems in the face of the pandemic [38], this is a very concerning finding in terms of overall population vulnerability to poor mental health.

We undertook stepwise model building to illustrate the sociodemographic and pandemic-related predictors of subjective wellbeing. In the pooled model and country models for Egypt and Morocco, women had significantly lower WHO-5 scores than men, consistent with our first hypothesis as well as previous literature finding a higher burden of poor mental health among women in the region [4]. Our finding of no gender gap in subjective wellbeing in Tunisia, Jordan and Sudan deserves future research on what factors may be protective of women’s mental health in these contexts.

We found that leaving employment had a significant negative association with wellbeing, consistent with literature from other countries during the pandemic [11,16,23], although a previous study of 18 MENA countries during the pandemic, based on non-representative data, did not find any significant association between employment status and changes in mental health [12]. The exceptions to this pattern were Jordan and Tunisia, where there was not a significant (although negative) effect in the multivariate models. Jordan forbade layoffs and had a relatively robust social safety net response for both formal and informal workers who experienced job losses [39], which may have cushioned the shock of the pandemic. This finding thus underscores the importance of effective policy responses in protecting wellbeing during crises. However, there were not significant differences between men and women in terms of the mental health impacts of leaving employment. H2 was therefore not supported.

Losses in income were negatively associated with wellbeing for both men and women, with no significant differences by sex, consistent with H3. This result has also been found in other studies from high- and middle-income countries [22,23]. Limitations in access to food were strongly negatively associated with wellbeing, with significant gender differences at higher levels of limitations. When food access was most restricted, men experienced more acute negative associations than women. This finding is at variance with H3. It is consistent, however, with a study from South Africa that found stronger associations between food insecurity and depressive symptoms among men than women during the pandemic. The author hypothesized that this finding may be related to anxiety around food access or shame around current methods for accessing food, such as relying on others [10]. These mechanisms may also be relevant in the MENA context, where men are expected to play the role of breadwinner [5]. It is interesting, however, that no similar association was seen for income loss, which suggests that the underlying stressor is more specific than general deprivation or decline in living standards. Rather, the barriers to food purchase experienced during the pandemic–some of which are related to supply factors rather than ability to pay–appear to be the driver of poorer wellbeing. At more severe levels of restriction, this stressor may have affected men more because of their expected role in providing for the household in the broader sense of meeting basic needs (e.g. finding ways to obtain food even in the face of supply-side constraints), and not only in terms of providing income.

We found no significant associations between social or government support (counter to H4), nor gender differences, except in Sudan where there was a negative relationship between government support and being female. This result, however, merits more research into the causal relationship; if support targets those who are worst off, associations may suffer from omitted variable bias. The heterogeneity of social assistance programs implemented in our study countries, and the short-term nature of many assistance schemes implemented during the pandemic, may also have made it difficult to detect any mental health effects of receiving assistance.

The results around helping with schoolwork showed a positive, but only sometimes significant relationship with wellbeing. Results varied substantially by country, with a negative significant interaction with being female in Jordan but a positive one in Egypt. These results are generally counter to H5 and our expectation that schooling modality would have no effect on men and a negative effect on women. They are also counter to the global literature finding negative gendered effects of school closures on women, which comes primarily from HICs [9,11,40]. One reason for the contrast in our findings may be the nature of alternative school modalities in our study countries and the relatively low quality of instruction. In Jordan, for example, young people reported spending less time on schoolwork during the pandemic [41]. If alternative schooling modalities effectively meant less schooling, the burden on women posed by helping with schoolwork may have been less than during ‘normal’ times.

Our results have a number of limitations, some of which highlight important areas for future research. The CMM data were designed to be representative of mobile phone users aged 18–64. The populations excluded from this frame may have had different experiences of wellbeing during the pandemic. Our data also miss the initial phase of the pandemic in early 2020, when closure measures were most severe in the study countries. We rely on respondents’ self-reports of pandemic-related responses, such as receipt of social assistance and alternative schooling modalities, which may suffer from response or recall biases. There are numerous factors related to alternative schooling modalities that are not captured in the data, such as the school-age children’s exact age and grade, attendance in public versus private schools, and quality and consistency of household internet access. These factors, in addition to issues around quality of education both during and prior to the pandemic, may contribute to the variability in our findings across countries. We also do not have a direct measure of time spent in care work during the pandemic, because this question was asked only of women in the CMM surveys and therefore cannot be used for a gender comparison. Specific studies on the schooling experiences during the COVID-19 pandemic are needed in order to better understand the impact of the disruptions on children and their parents in the region.

These limitations notwithstanding, our findings demonstrate the prevalence of poor subjective wellbeing as a health issue in the MENA region, one that appears to have been exacerbated by the pandemic. Further research is needed in particular into how social assistance–including food assistance–programs may be designed to mitigate not only the negative economic consequences of the pandemic but also potentially the negative mental health consequences. The lessons learned from the COVID-19 pandemic in this respect could be critical to future pandemic and other emergency response planning. Overall, despite the persistence of strongly gendered economic and care roles in the MENA region, the negative associations that we find between some pandemic-related socioeconomic changes and subjective wellbeing were less gendered than hypothesized. This may reflect a more generalized nature of socioeconomic stressors related to disruption in or loss of livelihoods during extraordinary times.

## Supporting information

S1 Table Waves and sample sizes of the COVID-19 MENA Monitor by country. (DOCX)

S2 Table COVID-19 related changes by country and sex, all respondents, CMM data. (DOCX)

S3 Table Summary statistics for the sociodemographic and household characteristics by country and sex, all respondents, CMM data. (DOCX)

S4 Table Associations (OLS regression coefficients) between subjective wellbeing and sex, (controlling for sociodemographic, and household structure variables) all respondents (H1). (DOCX)

S1 File Summary of social assistance programs and school closures during the COVID-19 pandemic in the study countries. (DOCX)

S2 File Fixed effects models results. (DOCX)
