# Supplementary material for: Impacts of the COVID-19 pandemic on subjective wellbeing in the Middle East and North Africa: A gender analysis
Source: PLoS One. 2023 May 31;18(5):e0286405. doi: 10.1371/journal.pone.0286405 (PMC10231778; doi:10.1371/journal.pone.0286405)
Supplement: S1 Table — (DOCX) [file pone.0286405.s001.docx]

**S1 Table: Waves and sample sizes of the COVID-19 MENA Monitor by country**

|  | **Wave** | | | | |  |
| --- | --- | --- | --- | --- | --- | --- |
| **Country** | **1: Nov. 2020** | **2: Feb. 2021** | **3: Apr. 2021** | **4: June 2021** | **5: Aug. 2021** | **Total** |
| **Jordan** |  | 2,549 |  | 2,503 | 2,573 | 7,625 |
| **Morocco** | 2,007 | 2,002 | 2,105 | 2,006 |  | 8,120 |
| **Sudan** |  |  | 2,400 |  | 2,001 | 4,401 |
| **Tunisia** | 2,000 | 2,077 | 2,057 | 2,009 |  | 8,143 |
| **Egypt** |  | 2,000 |  | 2,007 |  | 4,007 |
| **Total** | 4,007 | 8,628 | 6,562 | 8,525 | 4,574 | 32,296 |

Source: Constructed by the authors based on CMM data
