# Supplementary material for: Impacts of the COVID-19 pandemic on subjective wellbeing in the Middle East and North Africa: A gender analysis
Source: PLoS One. 2023 May 31;18(5):e0286405. doi: 10.1371/journal.pone.0286405 (PMC10231778; doi:10.1371/journal.pone.0286405)
Supplement: S2 Table — (DOCX) [file pone.0286405.s002.docx]

**S2 Table. COVID-19 related changes by country and sex, all respondents, CMM data.**

|  | **Jordan** | | **Morocco** | | **Sudan** | | **Tunisia** | | **Egypt** | |  |  |
| --- | --- | --- | --- | --- | --- | --- | --- | --- | --- | --- | --- | --- |
|  | **Men** | **Women** | **Men** | **Women** | **Men** | **Women** | **Men** | **Women** | **Men** | **Women** | **Total** | **N** |
| **COVID-19 changes applicable to all respondents** | |  |  |  |  |  |  |  |  |  |  |  |
| **Labor market status compared to Feb. 2020 (%)** | |  |  |  |  |  |  |  |  |  |  |  |
| Stayed employed | 53.9 | 11.6 | 55.1 | 15.8 | 42.0 | 7.8 | 63.5 | 18.8 | 72.5 | 15.5 | 38.4 | 13,476 |
| Stayed not employed | 28.1 | 79.4 | 16.8 | 68.7 | 14.7 | 63.6 | 15.8 | 65.9 | 11.4 | 74.5 | 41.2 | 12,158 |
| Left employment | 10.0 | 3.2 | 18.3 | 9.2 | 27.6 | 7.8 | 8.8 | 5.0 | 9.7 | 4.2 | 10.6 | 3,627 |
| Entered employment | 8.0 | 5.8 | 9.8 | 6.3 | 15.7 | 20.8 | 11.9 | 10.3 | 6.4 | 5.8 | 9.8 | 3,035 |
| **Last month income compared to Feb. 2020 (%)** | |  |  |  |  |  |  |  |  |  |  |  |
| Decreased by more than 25% | 26.5 | 25.9 | 51.0 | 52.6 | 52.2 | 49.5 | 26.6 | 21.7 | 21.7 | 22.3 | 35.0 | 11,518 |
| Decreased by 1-25% | 21.4 | 23.3 | 15.7 | 15.2 | 23.5 | 22.7 | 19.7 | 22.8 | 20.6 | 24.6 | 20.4 | 6,569 |
| Stayed the same | 43.7 | 43.7 | 27.8 | 28.7 | 10.7 | 19.4 | 44.2 | 47.9 | 46.9 | 45.9 | 36.7 | 11,500 |
| Increased | 8.3 | 7.2 | 5.5 | 3.5 | 13.6 | 8.4 | 9.5 | 7.6 | 10.8 | 7.2 | 7.9 | 2,688 |
| **Limitations in food access (mean number)** | 1.6 | 1.8 | 1.7 | 1.9 | 2.4 | 2.4 | 2.8 | 3.0 | 1.5 | 1.9 | 2.1 | 32,296 |
| **Regular government support (%)** |  |  |  |  |  |  |  |  |  |  |  |  |
| No | 61.0 | 58.7 | 87.6 | 82.7 | 85.2 | 94.5 | 85.9 | 83.6 | 20.7 | 21.7 | 71.9 | 23,364 |
| Yes | 39.0 | 41.3 | 12.4 | 17.3 | 14.8 | 5.5 | 14.1 | 16.4 | 79.3 | 78.3 | 28.1 | 8,932 |
| **Temporary (last month) government cash support (%)** | |  |  |  |  |  |  |  |  |  |  |  |
| No | 94.9 | 95.1 | 95.7 | 95.5 | 89.5 | 93.6 | 98.1 | 97.7 | 96.9 | 97.0 | 95.6 | 30,817 |
| Yes | 5.1 | 4.9 | 4.3 | 4.5 | 10.5 | 6.4 | 1.9 | 2.3 | 3.1 | 3.0 | 4.4 | 1,479 |
| **Last month social support (%)_** |  |  |  |  |  |  |  |  |  |  |  |  |
| No | 92.6 | 90.1 | 93.5 | 93.4 | 83.8 | 81.2 | 93.7 | 92.8 | 92.6 | 91.1 | 91.3 | 29,431 |
| Yes | 7.4 | 9.9 | 6.5 | 6.6 | 16.2 | 18.8 | 6.3 | 7.2 | 7.4 | 8.9 | 8.7 | 2,865 |
| N | 4,008 | 3,617 | 5,204 | 2,916 | 2,290 | 2,111 | 4,821 | 3,322 | 2,545 | 1,462 | 32,296 | **32,296** |
| **COVID-19 changes applicable to respondents with school age children in the household** | | | | |  |  |  |  |  |  |  |  |
| **Respondent helps with schoolwork (%)** |  |  |  |  |  |  |  |  |  |  |  |  |
| No | 70.3 | 39.2 | 85.2 | 85.7 | 59.5 | 57.0 | 45.1 | 27.3 | 39.2 | 18.2 | 56.5 | 9,817 |
| Yes | 29.7 | 60.8 | 14.8 | 14.3 | 40.5 | 43.0 | 54.9 | 72.7 | 60.8 | 81.8 | 43.5 | 8,135 |
| **Children in alternative schooling modality (%)** | |  |  |  |  |  |  |  |  |  |  |  |
| No | 9.5 | 7.5 | 50.5 | 50.3 | 40.4 | 37.2 | 6.4 | 6.7 | 4.0 | 5.5 | 23.4 | 3,887 |
| Yes | 90.5 | 92.5 | 49.5 | 49.7 | 59.6 | 62.8 | 93.6 | 93.3 | 96.0 | 94.5 | 76.6 | 14,065 |
| N | 2,469 | 2,318 | 2,983 | 1,580 | 1,235 | 939 | 2,414 | 1,594 | 1,459 | 961 | 17,952 | **17,952** |

Source: Constructed by the authors based on CMM data
