# Supplementary material for: Impacts of the COVID-19 pandemic on subjective wellbeing in the Middle East and North Africa: A gender analysis
Source: PLoS One. 2023 May 31;18(5):e0286405. doi: 10.1371/journal.pone.0286405 (PMC10231778; doi:10.1371/journal.pone.0286405)
Supplement: S3 Table — (DOCX) [file pone.0286405.s003.docx]

**S3 Table. Summary statistics for the sociodemographic and household characteristics by country and sex, all respondents, CMM data.**

|  | **Jordan** | | **Morocco** | | **Sudan** | | **Tunisia** | | **Egypt** | |  |  |
| --- | --- | --- | --- | --- | --- | --- | --- | --- | --- | --- | --- | --- |
|  | **Men** | **Women** | **Men** | **Women** | **Men** | **Women** | **Men** | **Women** | **Men** | **Women** | **Total** | **N** |
| **Age (mean)** | 37.0 | 36.6 | 28.2 | 36.2 | 34.1 | 30.0 | 39.4 | 37.2 | 36.8 | 35.4 | 36.7 | 32,296 |
| **Residence (%)** |  |  |  |  |  |  |  |  |  |  |  |  |
| Urban | 86.7 | 87.9 | 60.5 | 78.9 | 50.4 | 49.0 | 67.9 | 75.4 | 45.2 | 53.3 | 68.4 | 23,259 |
| Rural | 13.3 | 12.1 | 39.5 | 21.1 | 49.6 | 51.0 | 32.1 | 24.6 | 54.8 | 46.7 | 31.6 | 9,037 |
| **Highest level of education completed (%)** | |  |  |  |  |  |  |  |  |  |  |  |
| Less than basic | 16.7 | 21.0 | 71.8 | 63.5 | 24.0 | 24.3 | 52.0 | 49.0 | 25.6 | 27.9 | 41.1 | 7,203 |
| Basic | 32.2 | 22.2 | 16.9 | 17.7 | 19.6 | 15.5 | 15.2 | 16.0 | 7.6 | 7.9 | 18.1 | 5,947 |
| Secondary | 21.3 | 21.3 | 7.1 | 11.3 | 36.0 | 29.1 | 17.0 | 16.0 | 42.9 | 40.3 | 21.1 | 10,537 |
| Tertiary | 29.7 | 35.5 | 4.3 | 7.4 | 20.4 | 31.0 | 15.8 | 19.0 | 23.9 | 23.9 | 19.7 | 8,609 |
| **Marital status (%)** |  |  |  |  |  |  |  |  |  |  |  |  |
| Never married | 27.9 | 23.2 | 37.3 | 36.3 | 42.4 | 31.5 | 34.3 | 31.8 | 27.8 | 15.7 | 31.6 | 10,285 |
| Married | 70.5 | 69.3 | 61.0 | 53.4 | 57.1 | 62.0 | 64.3 | 61.1 | 70.3 | 73.9 | 63.9 | 20,520 |
| Widowed/divorced | 1.5 | 7.4 | 1.7 | 10.4 | 0.5 | 6.5 | 1.4 | 7.0 | 1.9 | 10.5 | 4.4 | 1,491 |
| **Refugee (%)** |  |  |  |  |  |  |  |  |  |  |  |  |
| No | 85.6 | 86.6 | - | - | - | - | - | - | - | - | 86.1 | 6,060 |
| Yes | 14.4 | 13.4 | - | - | - | - | - | - | - | - | 13.9 | 1,565 |
| **Total household size (mean)** | 5.5 | 5.2 | 6.1 | 5.6 | 7.4 | 6.4 | 4.5 | 4.3 | 4.6 | 4.4 | 5.4 | 32,296 |
| **Children under 6 in household (%)** | |  |  |  |  |  |  |  |  |  |  |  |
| No | 59.4 | 60.3 | 62.3 | 67.4 | 34.1 | 37.7 | 72.1 | 73.2 | 59.4 | 58.1 | 60.7 | 19,622 |
| Yes | 40.6 | 39.7 | 37.7 | 32.6 | 65.9 | 62.3 | 27.9 | 26.8 | 40.6 | 41.9 | 39.3 | 12,674 |
| **School age children in household (%)** | |  |  |  |  |  |  |  |  |  |  |  |
| No | 33.8 | 35.7 | 34.6 | 36.5 | 18.9 | 24.5 | 45.3 | 46.1 | 37.2 | 38.1 | 36.1 | 14,345 |
| Yes | 66.2 | 64.3 | 65.4 | 63.5 | 81.1 | 75.5 | 54.7 | 53.9 | 62.8 | 61.9 | 63.9 | 17,951 |
| N | 4,008 | 3,617 | 5,204 | 2,916 | 2,290 | 2,111 | 4,821 | 3,322 | 2,545 | 1,462 | 32,296 | 32,296 |

Source: Constructed by the authors based on CMM data
