# Supplementary material for: Impacts of the COVID-19 pandemic on subjective wellbeing in the Middle East and North Africa: A gender analysis
Source: PLoS One. 2023 May 31;18(5):e0286405. doi: 10.1371/journal.pone.0286405 (PMC10231778; doi:10.1371/journal.pone.0286405)
Supplement: S4 Table — (DOCX) [file pone.0286405.s004.docx]

**S4 Table. Associations (OLS regression coefficients) between subjective wellbeing and sex, (controlling for sociodemographic, and household structure variables) all respondents (H1)**

|  | **(1)** | **(2)** | **(3)** | **(4)** | **(5)** | **(6)** |
| --- | --- | --- | --- | --- | --- | --- |
| **Outcome variable:** Subjective well-being | **Pooled** | **Jordan** | **Morocco** | **Sudan** | **Tunisia** | **Egypt** |
| **Sex (ref: male)** |  |  |  |  |  |  |
| Female | -1.32* | 1.19 | -5.14*** | 0.63 | 0.48 | -3.18** |
|  | [-2.56 - -0.09] | [-0.65 - 3.04] | [-7.94 - -2.34] | [-4.03 - 5.29] | [-2.01 - 2.97] | [-5.21 - -1.16] |
| **Residence (ref: rural)** |  |  |  |  |  |  |
| Urban | -1.06 | -0.02 | -1.92 | 1.19 | 0.40 | -3.64*** |
|  | [-2.44 - 0.31] | [-2.94 - 2.89] | [-4.77 - 0.93] | [-3.07 - 5.44] | [-2.18 - 2.97] | [-5.60 - -1.69] |
| **Education (ref: less than basic)** |  |  |  |  |  |  |
| Basic | 2.22* | 2.76 | 1.27 | 0.40 | 6.31** | -3.43 |
|  | [0.26 - 4.18] | [-0.69 - 6.21] | [-2.17 - 4.71] | [-6.79 - 7.59] | [1.71 - 10.90] | [-6.95 - 0.09] |
| Secondary | 3.68*** | 5.43** | 5.59** | -0.92 | 5.47*** | -0.09 |
|  | [2.11 - 5.26] | [1.93 - 8.94] | [1.78 - 9.41] | [-6.98 - 5.13] | [2.71 - 8.23] | [-2.83 - 2.64] |
| Tertiary | 4.61*** | 9.39*** | 3.27 | -4.90 | 5.19*** | 1.27 |
|  | [2.96 - 6.26] | [5.80 - 12.98] | [-0.58 - 7.11] | [-11.33 - 1.53] | [2.14 - 8.24] | [-1.77 - 4.31] |
| **Marital status (ref: married)** |  |  |  |  |  |  |
| Never married | -0.25 | 2.01 | 1.68 | -5.16 | -1.20 | 2.72 |
|  | [-2.20 - 1.70] | [-1.05 - 5.07] | [-2.32 - 5.68] | [-10.92 - 0.60] | [-5.61 - 3.21] | [-0.71 - 6.15] |
| Widowed/divorced | -2.36 | -1.00 | -5.55* | -13.01** | 5.36* | -1.99 |
|  | [-4.98 - 0.26] | [-5.80 - 3.80] | [-10.84 - -0.26] | [-21.13 - -4.89] | [0.10 - 10.61] | [-7.67 - 3.69] |
| **Age** | 0.01 | -0.11 | 0.17* | 0.24 | -0.14 | 0.03 |
|  | [-0.06 - 0.07] | [-0.22 - 0.00] | [0.04 - 0.31] | [-0.02 - 0.51] | [-0.28 - 0.01] | [-0.09 - 0.16] |
| **Total household size** | -0.19 | -0.41 | -0.25 | 0.12 | -0.42 | -0.65 |
|  | [-0.52 - 0.15] | [-0.93 - 0.12] | [-0.82 - 0.33] | [-0.75 - 1.00] | [-1.18 - 0.35] | [-1.35 - 0.05] |
| **Children <6 in household** | -0.13 | 1.16 | -0.15 | 1.73 | -1.48 | -1.49 |
|  | [-1.56 - 1.30] | [-1.24 - 3.55] | [-3.09 - 2.78] | [-2.46 - 5.92] | [-4.80 - 1.84] | [-3.82 - 0.83] |
| **School age children in household** | -0.08 | 0.45 | -1.37 | -0.77 | 1.17 | 0.91 |
|  | [-1.41 - 1.26] | [-1.68 - 2.58] | [-4.30 - 1.56] | [-5.36 - 3.82] | [-1.63 - 3.98] | [-1.28 - 3.11] |
| **Refugee** |  | -0.17 |  |  |  |  |
|  |  | [-3.41 - 3.08] |  |  |  |  |
| **Wave (ref: Wave 1, Nov. 2020)** |  |  |  |  |  |  |
| Wave 2 (Feb. 2021) | -1.66 |  | 1.96 |  | 0.16 |  |
|  | [-4.39 - 1.07] |  | [-1.48 - 5.40] |  | [-1.98 - 2.30] |  |
| Wave 3 (April 2021) | 0.88 |  | 1.63 |  | 0.90 |  |
|  | [-1.31 - 3.07] |  | [-1.90 - 5.16] |  | [-1.27 - 3.06] |  |
| Wave 4 (June 2021) | -1.52 | 0.18 | -6.95*** |  | -1.51 | -0.47 |
|  | [-3.70 - 0.67] | [-1.44 - 1.81] | [-10.35 - -3.54] |  | [-3.68 - 0.67] | [-2.07 - 1.12] |
| Wave 5 (Aug. 2021) | -3.84** | -2.15* |  | -4.55* |  |  |
|  | [-6.50 - -1.17] | [-3.92 - -0.38] |  | [-8.56 - -0.54] |  |  |
| **Country (ref: Jordan)** |  |  |  |  |  |  |
| Morocco | 3.87* |  |  |  |  |  |
|  | [0.43 - 7.32] |  |  |  |  |  |
| Sudan | 10.43*** |  |  |  |  |  |
|  | [6.98 - 13.89] |  |  |  |  |  |
| Tunisia | -0.65 |  |  |  |  |  |
|  | [-2.88 - 1.59] |  |  |  |  |  |
| Egypt | 1.20 |  |  |  |  |  |
|  | [-0.71 - 3.11] |  |  |  |  |  |
| **Constant** | 39.94*** | 37.98*** | 40.25*** | 45.25*** | 42.71*** | 45.30*** |
|  | [35.40 - 44.47] | [31.03 - 44.93] | [32.42 - 48.07] | [31.66 - 58.85] | [34.76 - 50.66] | [38.81 - 51.79] |
| **Includes controls for country-wave interaction** | YES | N/A | N/A | N/A | N/A | N/A |
| **Observations** | 32,296 | 7,625 | 8,120 | 4,401 | 8,143 | 4,007 |
| **R-squared** | 0.03 | 0.04 | 0.03 | 0.05 | 0.03 | 0.02 |

Notes: 95% confidence intervals in brackets. * p<0.05, ** p<0.01, ***p<0.00
