# Supplementary material for: Impacts of the COVID-19 pandemic on subjective wellbeing in the Middle East and North Africa: A gender analysis
Source: PLoS One. 2023 May 31;18(5):e0286405. doi: 10.1371/journal.pone.0286405 (PMC10231778; doi:10.1371/journal.pone.0286405)
Supplement: S1 File — (DOCX) [file pone.0286405.s005.docx]

**S1 File. Summary of social assistance programs and school closures during the COVID-19 pandemic in the study countries**

All five countries included in this study implemented temporary social assistance programs during the height of the COVID-19 pandemic. Tunisia offered temporary cash transfers to the self-employed, temporary unemployment payments, and a one-time additional pension payment in mid-May 2020 of 100 TND, provided the retiree’s pension was less than 180 TND [1,2]. The household cash support was mainly during the spring lockdown (April-May 2020). Households eligible for subsidized health care (AMG II) collected 200 TND (USD 74) in each of April and May. Poor households already enrolled in the government cash transfer program (PNAFN) received 50 TND (USD 19) as cash assistance in April and May, in addition to their monthly aid [2].

In Morocco, laid off employees covered by social insurance (National Social Security Fund - CNSS) received a monthly cash transfer of 2,000 dirhams (200 USD) from April 2020 until March 2021 [3]. Meanwhile, starting April 2020, informal workers benefiting from the non-contributory health insurance (RAMED) collected between 800 and 1200 dirhams (USD 80-120) per month based on household composition. As to those not benefitting from RAMED, they were still able to obtain similar support by registering online [4].

Egypt expanded the targeted cash transfer social programs, Takaful and Karama, to reach more families [4]. It also launched a consumer spending initiative where manufacturers offered products at a discounted price, consumers could access low interest loans to pay for these goods, and households with ration cards received EGP 200 per household member (up to EGP 1000 - USD 64) per month to spend under this initiative [5]. Additionally, from May 2020 until March 2021, Egypt offered EGP 500 per month to irregular workers in severely hit sectors [4].

Jordan launched for three months, on the basis of the existing National Aid Fund (NAF) and Zakat Fund, JD 50-136 (USD 70-192) to 250,000 households delivered through e-wallets. Jordan also instituted and expanded a temporary cash transfer program for families, the self-employed, the unemployed, and daily workers through NAF [2]. In March 2021, Jordan extended the social welfare programs by mainly expanding the Takaful cash transfer program [4].

In Sudan, food and hygiene assistance, as well as cash cards, were provided to families during the initial three-month lockdown [6]. In February 2021, Sudan launched the Sudan Family Support Program (SFSP) or “Thamarat”, which provides eligible households with monthly cash transfers of USD 5, for a six-month period, based on the central bank of Sudan rate at the time. The implementation of Thamarat is rolled out in phases, with the aim of reaching 80% of families [7]. In November 2021, Thamarat suspended registration and direct cash payments until further notice, due to the World Bank, one of the main funders of the program, pausing its activities in Sudan [8]. Additionally, few people receive emergency assistance in the form of the “Selaaty” commodity support program [9].

In terms of school closures, all countries covered by this study suspended in-person education in schools and nurseries in March 2020. Jordan, Morocco, Egypt, and private schools in Tunisia adopted online learning, while Sudan and public schools in Tunisia remained closed. The 2020-2021 academic school year generally involved schools reopening for partial capacity in-person learning, occasionally switching to online classes for a few weeks (in Egypt) or to full in-person classes for a few months (in Tunisia and Morocco) depending on the number of COVID-19 cases in the country. Schools in Jordan remained mostly online while Sudanese schools were fully in-person. For the 2021-2022 academic year, schools reopened at full capacity for in-person learning in all these countries [10].

**References**

1. Hassen M, Marouani MA, Wojcieszynski E. Mitigation strategies in response to COVID-19 and inequalities in Tunisia. INCLUDE. 2021; 47.

2. Krafft C, Assaad R, Marouani MA. The Impact of COVID-19 on Middle Eastern and North African Labor Markets: Glimmers of Progress but Persistent Problems for Vulnerable Workers a Year into the Pandemic. In: Economic Research Forum (ERF) [Internet]. Jun 2021 [cited 7 Mar 2022]. Available: https://erf.org.eg/publications/the-impact-of-covid-19-on-middle-eastern-and-north-african-labor-markets-glimmers-of-progress-but-persistent-problems-for-vulnerable-workers-a-year-into-the-pandemic/

3. Kasraoui S. Coronavirus Pandemic: A Timeline of COVID-19 in Morocco. In: Morocco World News [Internet]. 2020 [cited 27 Jul 2021]. Available: https://www.moroccoworldnews.com/2020/03/296727/coronavirus-a-timeline-of-covid-19-in-morocco

4. IMF. Policy Responses to COVID19. In: International Monetary Fund [Internet]. 2021 [cited 6 Mar 2022]. Available: https://www.imf.org/en/Topics/imf-and-covid19/Policy-Responses-to-COVID-19

5. Enterprise. Egyptian government program to boost consumer spending goes live today. In: Enterprise [Internet]. 26 Jul 2020 [cited 7 Mar 2022]. Available: https://enterprise.press/stories/2020/07/26/egyptian-government-program-to-boost-consumer-spending-goes-live-today-19471/

6. UNICEF Sudan. COVID-19 Response 2020. In: UNICEF [Internet]. Mar 2021 [cited 7 Mar 2022]. Available: https://www.unicef.org/sudan/reports/covid-19-response

7. Thamarat. Thamarat Home. In: Thamarat [Internet]. 2021 [cited 6 Mar 2022]. Available: https://sfsp.gov.sd/

8. Thamarat. Clarification statement on the temporary cessation of payments and registrations. In: Thamarat [Internet]. 16 Nov 2021 [cited 6 Mar 2022]. Available: https://sfsp.gov.sd/sudan-family-support-programme-thamarat/

9. Krafft C, Assaad R. The impact of COVID-19 on the ability of households to meet their basic needs in Sudan. 2021; 2.

10. Krafft C, Selwaness I, Sieverding M. The Impact of the COVID-19 Pandemic on Women’s Care Work and Employment in the Middle East and North Africa. Cairo, Egypt: Economic Research Forum; 2022. Report No.: SWP 2022-5. Available: https://erf.org.eg/publications/the-impact-of-the-covid-19-pandemic-on-womens-care-work-and-employment-in-the-middle-east-and-north-africa/
