# Supplementary material for: Impacts of the COVID-19 pandemic on subjective wellbeing in the Middle East and North Africa: A gender analysis
Source: PLoS One. 2023 May 31;18(5):e0286405. doi: 10.1371/journal.pone.0286405 (PMC10231778; doi:10.1371/journal.pone.0286405)
Supplement: S2 File — (DOCX) [file pone.0286405.s006.docx]

**S2 File. Fixed effects models results**

The overall pattern of associations between COVID-19 related changes and subjective wellbeing largely remained the same in the fixed effects models, although the results became non-significant for a number of countries and types of changes. In terms of labor market status, the negative association between leaving employment and subjective wellbeing remained significant only in the country model for Morocco (Table B.5). Limitations on food access continued to be strongly associated with worse subjective wellbeing in the pooled model, for Morocco, and for some levels of limitation in Jordan, but the results for Tunisia and Egypt became non-significant. For income changes, the associations between decreased income and worse subjective wellbeing likewise continued to be negative but were only significant in the pooled model and for a decrease of 25% or more in Morocco. None of the interactions between food access or income changes and sex were significant in the fixed effects models.

There were also several results that became significant in the fixed effects models. In Morocco and Egypt, receipt of temporary (in the past month) government support was negatively associated with subjective wellbeing (-12.47 points and -9.06 points, respectively, p<0.05 in both cases) but the interaction with sex was positive and significant (25.49 points in Morocco, p<0.01 and 17.11 points in Egypt, p<0.05) (Table B.5). For the schooling results, whereas the positive association between helping with schoolwork and subjective wellbeing held for men in the pooled model, the negative interaction with sex became significant (-5.13 points, p<0.05) (Table B.6). Having children attending an alternative schooling modality was positively associated with subjective wellbeing in Egypt as well as Sudan in the fixed effects models, and negatively associated in Morocco and Tunisia.

**Table S2.1 Fixed-effect OLS regression coefficients between subjective wellbeing, sex and COVID-19 related changes, all respondents (H2-H4)**

|  | **(1)** | **(2)** | **(3)** | **(4)** | **(5)** | **(6)** |
| --- | --- | --- | --- | --- | --- | --- |
|  | **Pooled** | **Jordan** | **Morocco** | **Sudan** | **Tunisia** | **Egypt** |
| **Labor market change vs. Feb. 2020 (ref: stayed employed)** | | |  |  |  |  |
| Stayed not employed | -4.63 | -1.92 | -7.98 | -3.80 | -3.21 | 4.27 |
|  | [-10.22 - 0.96] | [-8.03 - 4.19] | [-24.44 - 8.49] | [-16.96 - 9.36] | [-9.37 - 2.94] | [-2.61 - 11.15] |
| Left employment | -3.26 | -2.37 | -8.02* | -0.06 | -1.64 | -0.10 |
|  | [-7.04 - 0.53] | [-6.90 - 2.15] | [-15.38 - -0.66] | [-11.65 - 11.53] | [-5.18 - 1.90] | [-6.31 - 6.12] |
| Stayed not employed # female | 3.47 | -1.40 | 7.01 | 5.81 | 0.96 | -4.17 |
|  | [-3.70 - 10.63] | [-11.90 - 9.10] | [-11.61 - 25.63] | [-13.84 - 25.46] | [-8.20 - 10.13] | [-14.35 - 6.01] |
| Left employment # female | 5.67 | 3.52 | 13.58 | 17.07 | -4.68 | -3.62 |
|  | [-2.56 - 13.90] | [-6.83 - 13.88] | [-1.08 - 28.24] | [-7.37 - 41.52] | [-12.32 - 2.96] | [-22.88 - 15.63] |
| **Changes in food access (ref: no change)** | |  |  |  |  |  |
| 1 limitation | -5.87** | -5.44* | -13.58*** | -5.77 | 2.17 | -0.65 |
|  | [-9.55 - -2.18] | [-9.59 - -1.29] | [-20.12 - -7.04] | [-26.51 - 14.98] | [-2.81 - 7.14] | [-5.65 - 4.34] |
| 2 limitations | -4.65* | -3.56 | -12.04*** | 10.66 | -0.64 | -1.28 |
|  | [-8.24 - -1.07] | [-8.00 - 0.88] | [-18.49 - -5.60] | [-9.20 - 30.53] | [-6.14 - 4.86] | [-8.21 - 5.66] |
| 3 limitations | -6.30*** | -3.26 | -14.46*** | 7.53 | -1.56 | -3.50 |
|  | [-10.02 - -2.59] | [-8.17 - 1.65] | [-22.04 - -6.88] | [-10.20 - 25.27] | [-6.51 - 3.38] | [-9.97 - 2.97] |
| 4 limitations | -5.76** | -1.63 | -16.29** | 5.21 | -0.65 | -0.50 |
|  | [-10.04 - -1.49] | [-8.22 - 4.96] | [-27.39 - -5.20] | [-12.87 - 23.30] | [-5.84 - 4.54] | [-8.19 - 7.19] |
| 5 limitations | -8.78*** | -11.08** | -19.56** | 16.21 | -3.57 | -8.04 |
|  | [-13.05 - -4.52] | [-17.91 - -4.25] | [-31.39 - -7.73] | [-3.92 - 36.34] | [-9.05 - 1.91] | [-19.83 - 3.76] |
| 1 limitation # female | 0.63 | 2.78 | 3.84 | -14.43 | -4.80 | -1.33 |
|  | [-4.69 - 5.94] | [-3.25 - 8.80] | [-8.05 - 15.74] | [-53.09 - 24.23] | [-13.63 - 4.03] | [-10.10 - 7.45] |
| 2 limitations # female | -0.21 | 1.81 | 5.09 | -21.37 | -6.00 | -3.90 |
|  | [-5.86 - 5.44] | [-4.82 - 8.44] | [-8.47 - 18.66] | [-56.60 - 13.87] | [-15.15 - 3.15] | [-14.61 - 6.81] |
| 3 limitations # female | -1.05 | 0.33 | 0.23 | -21.05 | -6.56 | -3.34 |
|  | [-7.03 - 4.94] | [-6.90 - 7.56] | [-13.07 - 13.53] | [-56.41 - 14.31] | [-17.75 - 4.63] | [-12.87 - 6.19] |
| 4 limitations # female | 1.23 | 3.83 | 14.72 | -25.23 | -7.89 | -6.07 |
|  | [-4.96 - 7.43] | [-5.52 - 13.18] | [-0.61 - 30.06] | [-62.49 - 12.03] | [-17.84 - 2.07] | [-18.08 - 5.93] |
| 5 limitations # female | 2.63 | 6.42 | 8.64 | -22.45 | -4.82 | 5.23 |
|  | [-4.19 - 9.46] | [-6.01 - 18.86] | [-10.90 - 28.19] | [-59.96 - 15.06] | [-15.66 - 6.02] | [-9.44 - 19.90] |
| **Income changes vs. Feb. 2020 (ref: stayed the same)** | | |  |  |  |  |
| Decreased by more than 25% | -4.49** | -2.80 | -7.86* | -4.92 | -2.14 | -2.73 |
|  | [-7.24 - -1.74] | [-7.00 - 1.39] | [-14.83 - -0.89] | [-19.97 - 10.14] | [-4.89 - 0.62] | [-9.09 - 3.64] |
| Decreased by 1-25% | -3.84** | -2.07 | -6.92 | -9.68 | -1.37 | -4.46 |
|  | [-6.64 - -1.05] | [-6.05 - 1.90] | [-15.05 - 1.21] | [-23.32 - 3.95] | [-4.28 - 1.53] | [-10.21 - 1.29] |
| Increased | 0.36 | 1.44 | -2.30 | 5.67 | -0.61 | -6.55 |
|  | [-3.87 - 4.59] | [-3.54 - 6.42] | [-16.74 - 12.14] | [-9.97 - 21.30] | [-4.48 - 3.26] | [-14.21 - 1.10] |
| Decreased by more than 25% # female | 3.33 | 2.57 | 5.36 | -0.74 | 0.06 | 3.18 |
|  | [-0.68 - 7.34] | [-2.87 - 8.00] | [-6.14 - 16.85] | [-29.67 - 28.18] | [-4.49 - 4.61] | [-5.74 - 12.11] |
| Decreased by 1-25% # female | 1.70 | 0.37 | 2.31 | 0.35 | -0.26 | 4.12 |
|  | [-2.41 - 5.81] | [-5.20 - 5.95] | [-10.37 - 14.99] | [-31.51 - 32.22] | [-6.01 - 5.50] | [-3.19 - 11.43] |
| Increased # female | 1.20 | 4.25 | 1.44 | -14.79 | 0.11 | 10.04 |
|  | [-4.94 - 7.33] | [-4.72 - 13.22] | [-19.47 - 22.35] | [-42.57 - 12.99] | [-6.49 - 6.71] | [-2.22 - 22.30] |
| **Receipt of transfers** |  |  |  |  |  |  |
| Temporary government support | -1.60 | 3.83 | -12.47* | 3.71 | 1.00 | -9.06* |
|  | [-6.55 - 3.35] | [-1.45 - 9.11] | [-24.39 - -0.55] | [-7.65 - 15.08] | [-6.28 - 8.28] | [-17.02 - -1.10] |
| Temporary government support # female | 6.22 | -7.06 | 25.49** | -3.73 | 10.98 | 17.11* |
|  | [-0.86 - 13.31] | [-15.57 - 1.46] | [9.40 - 41.57] | [-26.25 - 18.79] | [-1.15 - 23.12] | [3.64 - 30.58] |
| Last month social support | 0.96 | -0.93 | 8.30 | -9.80 | -1.34 | 4.07 |
|  | [-3.68 - 5.61] | [-5.76 - 3.91] | [-4.86 - 21.47] | [-21.48 - 1.89] | [-6.64 - 3.95] | [-1.34 - 9.49] |
| Last month social support # female | 2.10 | -1.64 | -0.67 | 12.78 | 6.48 | -5.92 |
|  | [-3.93 - 8.13] | [-7.72 - 4.43] | [-15.68 - 14.33] | [-9.25 - 34.81] | [-4.62 - 17.58] | [-13.53 - 1.69] |
| Constant | 49.38*** | 42.41*** | 54.81*** | 56.63*** | 44.50*** | 43.52*** |
|  | [46.01 - 52.74] | [38.35 - 46.46] | [46.89 - 62.73] | [37.51 - 75.74] | [39.72 - 49.28] | [39.15 - 47.90] |
| Includes controls for survey wave | YES | YES | YES | YES | YES | YES |
| Includes controls for country-wave interaction | YES | N/A | N/A | N/A | N/A | N/A |
| Observations | 32,275 | 7,625 | 8,099 | 4,401 | 8,143 | 4,007 |
| R-squared | 0.04 | 0.03 | 0.10 | 0.11 | 0.02 | 0.04 |
| Number of unique individuals | 20,241 | 4,549 | 5,124 | 3,949 | 3,495 | 3,124 |

Notes: 95% confidence intervals in brackets. * p<0.05, ** p<0.01, ***p<0.001

**Table S2.2 Fixed-effect OLS regression coefficients between subjective wellbeing and changes in schooling modalities, respondents with school aged children (H5)**

|  | **(1)** | **(2)** | **(3)** | **(4)** | **(5)** | **(6)** |
| --- | --- | --- | --- | --- | --- | --- |
|  | **Pooled** | **Jordan** | **Morocco** | **Sudan** | **Tunisia** | **Egypt** |
| **Respondent helps with school work** | 2.91* | 2.67 | 5.52 | -4.97 | 2.33 | 0.49 |
|  | [0.14 - 5.68] | [-1.81 - 7.16] | [-1.45 - 12.49] | [-17.17 - 7.24] | [-1.16 - 5.83] | [-4.76 - 5.75] |
| Respondent helps with school work # female | -5.13* | -8.08** | -6.29 | -8.83 | 0.82 | -3.13 |
|  | [-9.61 - -0.66] | [-14.16 - -1.99] | [-18.98 - 6.39] | [-29.99 - 12.32] | [-5.94 - 7.59] | [-12.09 - 5.84] |
| **Children in alternative schooling modality** | -2.92 | -2.15 | -8.63* | 22.14*** | -8.78** | 9.88* |
|  | [-7.42 - 1.57] | [-9.12 - 4.82] | [-15.56 - -1.71] | [9.32 - 34.95] | [-15.17 - -2.40] | [0.93 - 18.83] |
| Children in alternative schooling modality # female | 1.69 | 5.40 | 1.14 | -10.57 | 8.16 | -4.30 |
|  | [-3.89 - 7.26] | [-5.18 - 15.99] | [-7.00 - 9.27] | [-30.17 - 9.02] | [-2.06 - 18.37] | [-17.24 - 8.63] |
| **Constant** | 51.00*** | 41.80*** | 60.56*** | 45.04*** | 49.13*** | 38.02*** |
|  | [45.83 - 56.17] | [35.34 - 48.27] | [50.54 - 70.58] | [29.72 - 60.36] | [40.40 - 57.86] | [28.57 - 47.47] |
| Includes controls for other COVID-19 changes | YES | YES | YES | YES | YES | YES |
| Includes controls for survey wave | YES | YES | YES | YES | YES | YES |
| Includes controls for country-wave interaction | YES | N/A | N/A | N/A | N/A | N/A |
| Observations | 17,937 | 4,787 | 4,548 | 2,174 | 4,008 | 2,420 |
| R-squared | 0.05 | 0.05 | 0.13 | 0.24 | 0.07 | 0.07 |
| Number of unique individuals | 11,570 | 2,891 | 2,996 | 1,909 | 1,877 | 1,897 |

Notes: 95% confidence intervals in brackets. * p<0.05, ** p<0.01, ***p<0.001.
